# Lipid Profile and Vascular Remodelling in Young Dyslipidemic Subjects Treated with Nutraceuticals Derived from Red Yeast Rice

**DOI:** 10.1155/2021/5546800

**Published:** 2021-04-22

**Authors:** Massimo Puato, Alberto Zambon, Chiara Nardin, Elisabetta Faggin, Raffaele Pesavento, Alice Spinazzè, Paolo Pauletto, Marcello Rattazzi

**Affiliations:** ^1^Medicina, Ospedale di Mirano, Mirano, Italy; ^2^Department of Medicine (DIMED), University of Padova, Italy; ^3^Medicina Generale I^, Ca' Foncello Hospital, Treviso, Italy; ^4^ORAS Rehabilitation Hospital, Motta di Livenza, Treviso, Italy

## Abstract

**Background and Aims:**

A relevant role is emerging for functional foods in cardiovascular prevention. The aim of this study was to assess the effect of a nutraceutical multitargeted approach on lipid profile and inflammatory markers along with vascular remodelling in a cohort of dyslipidemic subjects without history of cardiovascular (CV) disease.

**Methods and Results:**

We enrolled 25 subjects (mean age 48.2 years) with low to moderate CV risk profile and total cholesterol (TC) levels between 150 and 250 mg/dl. The patients were assigned to receive for one year a tablet/die of a nutraceutical combination containing red yeast rice (RYR) extract (Monacolin 3 mg/tablet) and coenzyme Q10 (30 mg/tablet). Treatment with the nutraceutical compounds led to a significant reduction of TC (from 227 to 201 mg/dl, *p* < 0.001), LDL-c (from 150 to 130 mg/dl, *p* = 0.001), triglycerides (from 121 to 109 mg/dl, *p* = 0.013), non-HDL-cholesterol (from 168 to 141 mg/dl, *p* < 0.001), hs-CRP (from 1.74 to 1.20 mg/l, *p* = 0.015), and osteoprotegerin (from 1488 to 1328 pg/ml, *p* = 0.045). Levels of HDL-c, Lp(a), glucose, liver enzyme, CPK, or creatinine did not change over time. An ultrasound study was performed to assess changes in mean carotid intima-media thickness (IMT) and maximum IMT (M-MAX) as well as modification in local carotid stiffness by means of determining the carotid compliance coefficient (CC) and distensibility coefficient (DC). At the end of the treatment, we observed small but significant reductions in both mean-IMT (from 0.62 to 0.57 mm, *p* = 0.022) and M-MAX (from 0.79 to 0.73 mm, *p* = 0.002), and an improvement in carotid elasticity (DC from 22.4 to 24.3 × 10^−3^/kPa, *p* = 0.006 and CC from 0.77 to 0.85 mm^2^/kPa, *p* = 0.019).

**Conclusions:**

A long-term treatment with a combination of RYR and coenzyme Q10 showed lipid-lowering activity along with a reduction of inflammatory mediators and an improvement of vascular properties in young subjects with a low-to-moderate CV risk profile.

## 1. Introduction

Atherosclerosis represents a leading cause of cardiovascular (CV) morbidity and mortality. Hypercholesterolemia is one of the most common modifiable CV risk factors, and it is well established that lowering low-density lipoprotein cholesterol (LDL-c) is associated with a significant decrease in CV diseases [1–4]. Although a combination of effective pharmacological approaches is available and LDL-c target levels have been well defined in the current European Society of Cardiology/European Atherosclerosis Society (ESC/EAS) Guidelines [5], low-risk patients, usually characterized by cholesterol levels slightly above the normal range and limited burden of CV events, are often suboptimally treated. This is probably due to a combination of misperception of the role of CV risk factors; poor compliance to lifestyle measures; and drug adverse effects such as statin-associated muscle symptoms (SAMS), reported as statin intolerance, which make treatment in this group of subjects a matter of debate and often a clinical challenge. In the recent years, a relevant role is emerging for functional foods with lipid-modifying effects and the potential to be considered for CV prevention in selected populations, especially in primary CV patients at low/intermediate CV risk and with mild to moderate hypercholesterolemia [6, 7].

Red yeast rice RYR is a nutraceutical, traditionally used in Chinese medicine, obtained by the fermentation of rice (*Oryza sativa*) as result of a yeast (in general *Monascus purpureus*, *M. pilosus*, *M. floridanus*, *M. ruber*, and *Pleurotus ostreatus*), whose typical red colouration is due to the presence of pigments that are by-products of the fermentative metabolism process. It contains monacolins, statin-like molecules able to inhibit 3-hydroxyl-3-methyl-glutaryl-CoA (HMG-CoA) reductase [8]. Many studies have documented the efficacy of RYR as a lipid-lowering agent, in both people at low and high CV risk [7]. In a large meta-analysis of approximately 9700 patients, RYR led to a mean reduction in total cholesterol (TC), LDL-c, and triglycerides (TG) and a mild increase in high-density lipoprotein cholesterol (HDL-c) values [9]. In a double-blind multicenter trial in China, RYR was associated with a significant decrease in the incidence of major coronary events in 4870 patients with previous myocardial infarction, after 4.5 years follow-up [10].

Coenzyme Q10 (CoQ10) is an antioxidant agent present in the membranes of most human cells. It is a crucial component of the mitochondrial respiratory chain accepting or losing two electrons to form hydroquinone or benzoquinone, respectively [11]. CoQ10 has been reported to have beneficial effects on lipid and metabolic profiles [12], together with antiatherosclerotic properties [13, 14].

Although the use of RYR and CoQ10 is supported by randomized controlled trials and meta-analysis, data regarding their effects on CV prevention and vascular remodelling are lacking.

The aim of this study was to assess the effect of a long-term nutraceutical multitarget approach on lipid profile, inflammatory markers, and vascular remodelling in primary CV prevention in a setting of controlled dietary habits. The nutraceutical combination used in this study consisted of a single pill containing 333 mg of RYR, equivalent to 10 mg of Monacolin K and 30 mg of CoQ10.

## 2. Methods

### 2.1. Participants

Thirty subjects aged 30-65 years referred to Clinica Medica III at Padova University Hospital (Azienda Ospedaliera Universitaria di Padova, Padova, Italy) and were enrolled between May 2016 and December 2017. Eligibility criteria for the study were as follows: TC > 150 mg/dl and <250 mg/dl; TG > 150 mg/dl and <500 mg/dl; fasting glucose < 126 mg/dl; and stable antihypertensive treatment (at least for 6 months) in the presence of hypertension. Exclusion criteria were as follows: TC > 250 mg/dl and/or TG > 500 mg/dl and/or previous statin therapy (last 6 months); treatment with hypoglycemic agents and/or fasting glucose > 126 mg/dl; chronic gastrointestinal disorders; abnormal renal function (eGFR < 60 ml/min/1.73 m^2^); concomitant treatment with drugs potentially interfering with glucose and lipid metabolism; history of CV diseases; proven intolerance to any component of the nutraceutical product; and pregnancy or breastfeeding. The study was approved by the local Research Ethics Committee and registered with https://ClinicalTrial.org (CT Identifier: NCT04433429). All patients gave written informed consent.

### 2.2. Study Design

All participants were treated with Liposcudil Plus (one pill/day, containing 333 mg of RYR, equivalent to 10 mg of Monacolin K and 30 mg of CoQ10, Piam Farmaceutici, Italy) together with a controlled Mediterranean diet training and support. Patients with stable dietary habits according to guidelines for CVD prevention [15] since at least 6 months and who did meet their individual lipid goals were enrolled in this study. During the study period, patients were advised to maintain such a diet unchanged. Study assessments were performed at baseline and after 12 months (supplementary figure (available here)). At baseline, lifestyle and medical history were recorded; anthropometric measurements were performed, and body mass index (BMI) was calculated. Systolic, diastolic blood pressure, and resting heart rate were taken after three readings over a five-minute period. All subjects underwent blood sampling and carotid intima-media thickness (IMT) evaluation; carotid compliance coefficient (CC) and distensibility coefficient (DC) were derived. Women had a pregnancy test performed at recruitment. After 6 months, all participants received a phone call to verify the adherence to therapy and dietary habits. After 12 months, they underwent a new clinical visit, blood tests, IMT, CC, and DC measurements. 25 subjects (11 males and 14 females) completed the treatment and performed all the visits.

### 2.3. Biochemical Measurements

Blood samples were collected from the antecubital vein under fasting conditions and stored at -80° C until use. Blood count, lipid profile (TC, LDL-c, HDL-c, and TG), serum glucose, and renal and hepatic function were assessed using standard laboratory techniques. Lp(a) levels were evaluated using a standard immunochemical-based assay with reference ranges < 30 mg/dl or <75 nmol/l [16, 17].

The LDL peak buoyancy and cholesterol distribution across the lipoprotein classes were assessed by density gradient ultracentrifugation (DGUC) for apo B containing lipoproteins, optimized for LDL particles. Thirty-seven 0.45 ml fractions were then collected from the bottom of the centrifuge tube. Cholesterol was measured in each fraction. The relative flotation rate (Rf), which characterizes LDL peak buoyancy, was obtained by dividing the fraction number containing the LDL-cholesterol peak by the total number of fractions collected. Each lipoprotein subclass elution range was defined as previously published [18]. Circulating levels of osteoprotegerin (OPG) were assessed by using dedicated ELISA kits (R&D System), whereas serum levels of high-sensitivity CRP (hs-CRP) have been measured through a sensitive immunonephelometric method (Dade Behring).

### 2.4. Ultrasound Examination of Carotid Arteries

Carotid ultrasound examinations were performed as previously described [19], using the Aspen Advanced Ultrasound System (Acuson, Mountain View, CA, USA) equipped with a linear probe (7–10 MHz). The procedure was carried out according to the Mannheim Intima-Media Thickness Consensus [20]. Briefly, all subjects were examined in the same room in dim light, lying comfortably in a supine position. The right and left carotid arteries of each subject were examined by the same sonographer. IMT, defined as the distance between the lumen-intima and the media-adventitia interfaces, was measured at end diastole in the far wall of the right and left sides of the common carotid artery, the bulb, and the internal carotid artery [21]. IMT measurements were expressed as cumulative mean of mean-IMT (mean-IMT) and as cumulative mean of maximum IMT (M-MAX) recorded in each vascular segment. The within- and between-observer measurement reproducibility values of IMT measurements on separate visits displayed a coefficient of variation of 4.3%. Local carotid stiffness was assessed by a B-mode-based system coupled with dedicated software and expressed as CC and DC [22]. The reproducibility of the local carotid stiffness assessment displayed a coefficient of variation of 4.4% and 9.6% for CC and DC, respectively. The Bland-Altman statistics confirmed the good reproducibility of measurements.

### 2.5. Statistical Analysis

All the clinical, biochemical, and ultrasound data were collected at baseline and after 12 months of treatment and were presented as mean ± standard deviation (SD). To estimate the effect of the treatment, the statistical analysis was performed by using paired*t*-tests in cases of variables normally distributed, or the Wilcoxon signed-rank test in cases of variables without normal distribution. Mean and 95% CI have been calculated for each DGUC lipoprotein fraction at baseline and follow-up. LDL Rf values and cholesterol levels in each DGUC fraction on and off treatment were analyzed by using the paired Student *t*-test, or the Wilcoxon signed-rank test if not normally distributed. Significance was considered for *p* < 0.05. All statistical the analyses were performed by using SPSS statistics 25.0 (IBM, USA).

## 3. Results

### 3.1. Lipid Profile and Biochemical Data

**Figure 1:**
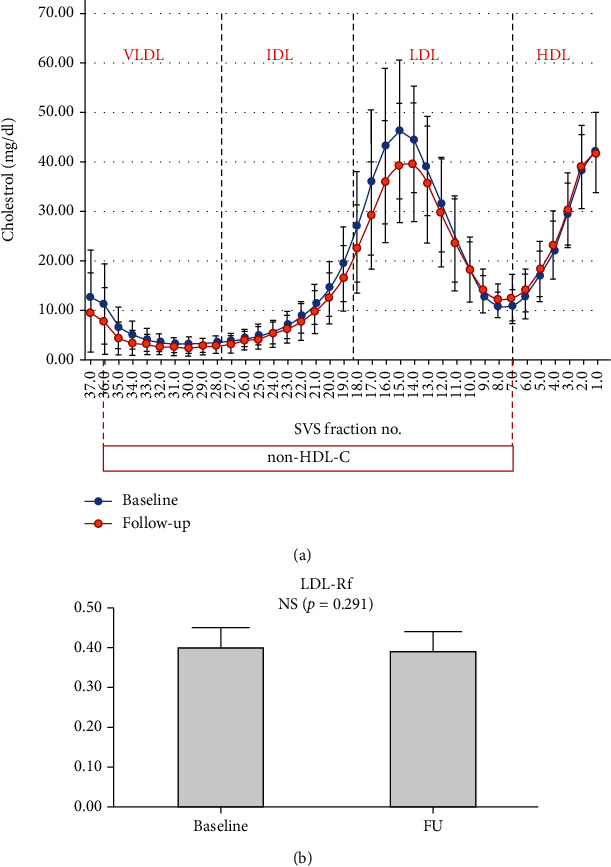
Effect of treatment on LDL density and distribution of lipoprotein classes. Density gradient ultracentrifugation (DGUC) (a) and relative flotation rate (Rf) (b) were performed at baseline and after 12 months of treatment to determine changes in lipoprotein class distribution and LDL peak buoyancy, respectively. We found no significant modification in LDL density (b), whereas DGUC analysis showed a significant decrease in the LDL-c subfractions together with a trend of reduction in TG-rich lipoproteins (a) (VLDL: very low-density lipoproteins; IDL: intermediate-density lipoproteins; LDL: low-density lipoproteins; HDL: high-density lipoproteins; Non-HDL-c: non-HDL cholesterol; FU: follow-up).

Twenty-five participants (11 males and 14 females, mean age 48.2 ± 11.9 years) with low-moderate CV risk (calculated 10-year mean SCORE risk of 0.8%) completed a 12-month treatment with Liposcudil Plus. Anthropometric and biochemical data of the population at baseline and at the end of study period are summarized in Table 1. Overall, the treatment was well tolerated, and none of the patients reported myalgia or significant adverse events. While the treatment did not induce significant changes in BMI, blood pressure, and heart rate, we observed a significant reduction in TC (*p* < 0.001), LDL-c (*p* = 0.001), and TG levels (*p* = 0.013) as well as non-HDL-c (*p* < 0.001). On the contrary HDL-c and Lp(a) levels were not affected by the treatment (*p* = 0.6 and *p* = 0.4, respectively). We also investigated by DGUC the effect of the treatment on lipoprotein subclass distribution (Figure 1(a)). The analysis confirmed a significant decrease in the LDL-c subfractions without a major shift in the density distribution of the LDL subclasses, whereas no changes were found in the levels of HDL-c subclasses. Of interest, and in line with the findings on circulating TG levels, this analysis also showed a trend towards a reduction of cholesterol transported in TG-rich lipoproteins (very low-density lipoproteins (VLDL)). Finally, the value of flotation rate values was not significantly modified by the treatment, suggesting that the nutraceutical compound did not affect LDL density distribution while decreasing LDL-c levels (Figure 1(b)).

**Figure 2:**
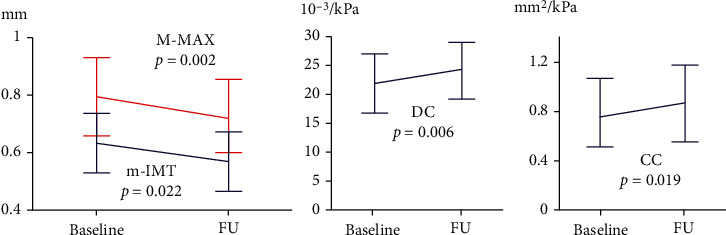
Ultrasound parameters at baseline and follow-up. Carotid ultrasound examinations were performed at enrollment and after 12 months. At the end of the treatment, we observed a small but significant reduction in both mean-IMT and M-MAX, and improvement in carotid elasticity (m-IMT: cumulative mean of mean IMT recorded in each carotid artery segment on both sides; M-MAX: cumulative mean of maximum IMT recorded in each carotid artery segment on both sides; DC: distensibility coefficient; CC: compliance coefficient).

As for the other parameters under investigation, no differences, before and after treatment, were found in fasting glucose and renal/liver function. In particular, the treatment did not induce significant changes in transaminase and CPK levels. On the other hand, after 12 months of treatment, we observed a small but significant decrease in the circulating levels of two markers of inflammation (hs-CRP *p* = 0.015 and OPG *p* = 0.045) (Figure 2).

### 3.2. Ultrasound Study

Results of the ultrasound analyses were available for all the 25 patients who completed the study. We observed that the treatment with Liposcudil Plus was followed by a small but significant reduction in carotid IMT. This reduction was found for both mean-IMT (0.62 ± 0.13 mm vs. 0.57 ± 0.12 mm, *p* = 0.022) and M-MAX (0.79 ± 0.16 mm vs. 0.73 ± 0.13 mm, *p* = 0.002), suggesting a positive effect of the nutraceutical compound on the early stages of the atherosclerotic process. Moreover, at the end of the treatment, we observed a modest although significant improvement in carotid elasticity (DC from 22.4 ± 5.3 to 24.3 ± 5.5 10 − 3/kPa, *p* = 0.006 and CC from 0.77 ± 0.30 to 0.85 ± 0.34 mm^2^/kPa, *p* = 0.019) (Figure 2).

## 4. Discussion

Our major findings were that treatment with Liposcudil Plus, a combination of RYR delivering 10 mg Monacolin K/day and 30 mg/day of CoQ10, was associated with an improvement in lipid and inflammatory profile, together with an overall positive effect on vascular remodelling.

In the current study, the combination of RYR and CoQ10 showed a significant reduction in TC, LDL-c, non-HDL-c, and TG levels, while HDL-c did not change over time. Although LDL-c significantly decreased with Liposcudil Plus, LDL subfraction distribution across a continuous density range and LDL peak density was not affected. Moreover, it was safe and well tolerated, without increasing transaminase and CPK levels. The RYR lipid-lowering activity, alone or in combination with other components like berberine and policosanol [23], has been well established in many clinical trials. In a large meta-analysis of approximately 9700 patients, RYR led to a mean decrease in TC, LDL-c, and TG and a mild increase in HDL-c values [9]. More recently, Becker et al. demonstrated a LDL-c decrease of 43 mg/dl and 35 mg/dl from baseline at weeks 12 and 24, respectively, in the RYR treatment group, versus a decrease of 11 mg/dl and 15 mg/dl from baseline at weeks 12 and 24, respectively, in the placebo group, without increasing CPK or pain levels [24]. Relatively few studies have investigated the role of CoQ10 as a lipid-lowering agent, and data are controversial. In a meta-analysis, including seven trials and involving 356 patients, CoQ10 alone did not alter LDL-c, HDL-c, and blood pressure values but produced a weak TG level-lowering effect [25]. In another randomized, double-blinded, placebo-controlled trial, CoQ10 was associated with a significant decrease of non-HDL-c values [12]. Only one previous study investigated the effect of RYR and CoQ10 supplementation in hypertensive and hypercholesterolemic subjects with metabolic syndrome. The authors found a greater reduction in blood pressure, TC, LDL-c, TG, and serum glucose levels in the treatment group compared with the placebo group after 2 months [26]. Although these studies were performed on a population with different characteristics, our results are in accordance with the abovementioned trial.

In the current study, treatment with Liposcudil Plus decreased inflammatory markers such as hs-CRP and OPG and showed a positive effect on vascular remodelling through its lipid-lowering and anti-inflammatory activities. Some randomized placebo-controlled clinical trials support the beneficial effects of RYR and CoQ10 on CV risk. RYR has been associated with improvement in endothelial function and reactivity [27] and carotid-femoral pulse wave velocity [28], along with a reduction in hs-CRP, interleukin-6, and interleukin-1 levels [27], with a good tolerability profile. Our findings are in line with these previous reports confirming that, similarly to statins, the lipid level reduction achieved with RYR is accompanied by a significant reduction in hs-CRP levels. On the other side, CoQ10 supplementation has been shown to not affect hs-CRP, but to increase serum total antioxidant capacity [12] and decrease oxidative stress in low-density lipoproteins [29] and endothelial cells [30]. In our study, treatment with Liposcudil also produced a significant reduction in OPG over time. The latter is a circulating marker that has been strongly associated with an increased risk of atherosclerosis progression and cardiovascular disease [31, 32]. Nevertheless, its actual role in the pathogenesis of the vascular damage is still controversial [33, 34]. Several studies clearly showed that inflammation, together with other clinical determinants, can play a relevant role in driving the residual cardiovascular risk observed among patients who undergo CV events despite reaching optimal therapeutic targets [35, 36]. Theoretically, primary intervention strategies characterized by anti-inflammatory proprieties, including nutraceutical compounds, could exert an additional protective effect on the progression of cardiovascular damage. Of interest, our study showed a significant effect of Liposcudil in reducing plasma TG levels mainly through a reduction in VLDL subclasses as suggested by DGUC analysis. This effect could be due to an additive effect of RYR and CoQ10 in reducing TG levels and significantly contributing to the anti-inflammatory activity of the nutraceutical compound. In fact, several lines of research clearly showed that TG-rich lipoproteins (including remnants) can have a detrimental effect on the inflammatory processes linked to atherosclerosis progression and complication [37].

Recent evidence focused on the clinical relevance of lifetime exposure to LDL-c levels as a key parameter to evaluate the individual risk of CVD events [38]. The concept that “The lower the LDL-c the better” is a strong, evidence-based fact; however, based on the evidence published in the past five years, the updated version of this concept suggests “The longer the lower LDL-c is maintained the better”. This is a crucial shift in our way to approach CVD prevention. Small differences in LDL-c, i.e., 25-30 mg/dl (0.6-0.8 mmol/l), maintained over a long period (12-15 years) will likely result in a CVD risk reduction comparable with a 5-year moderate- to high-intensity statin approach. Nutraceuticals, namely, RYR, can be envisioned as a support to a healthy lifestyle to achieve, in a large number of patients at low-intermediate CV risk, a remarkable CV event reduction when started earlier in life and maintained over the years [7]. At the end of the study, we did not observe a significant effect of RYR/CoQ10 on the circulating levels of Lp(a). This lipid particle is a well-known independent predictor of future CV events in both the primary and secondary prevention settings [39]. Unfortunately, current lipid-lowering medications available in the clinical practice have limited effects on circulating Lp(a), and it is unclear whether its reduction can significantly impact on the CV risk profile [39]. The lack of effect of RYR on Lp(a) levels was not unexpected, and new drugs are approaching the clinical ground to verify whether the pharmacological modulation of this particle could play a role in halting vascular disease progression and reducing the risk of CV events [40].

To the best of our knowledge, this is the first study to investigate the effect of this nutraceutical combination on vascular remodelling. Several studies showed that carotid IMT is an independent predictor of future CV events, although its relevance for the cardiovascular risk reclassification of the patients is still a matter of debate [41, 42]. Nevertheless, there is a substantial agreement that an increase in carotid IMT is indicative of an early atherosclerotic vascular damage, and several intervention strategies known to reduce CV risk have shown to halt the progression of carotid remodelling [43]. In the present study, we found that both mean-IMT and M-MAX were slightly reduced by treatment and that carotid elasticity was significantly improved. This effect could be driven by the synergistic lipid-lowering, anti-inflammatory, and antioxidant activity of RYR and CoQ10. However, other clinical studies are needed to confirm the positive effects on vascular remodelling observed in our population.

The small size of population and the lack of a placebo-controlled group are the mean limitations of the current study. Moreover, as all subjects followed a controlled dietary habit, we are not able to distinguish the role of diet from the effects of the treatment.

In conclusion, treatment with Liposcudil Plus was safe and well tolerated and produced an improvement in the lipid and inflammatory profile, along with a protective vascular remodelling. A nutraceutical multitarget approach could be useful in reducing both cholesterol values and inflammatory risk and could be started early in subjects with low-to-moderate CV risk. Further randomized, double-blinded, placebo-controlled trials are required before the nutraceutical way can become the routine approach to moderate dyslipidemia control in routine clinical practice.

## Figures and Tables

**Table 1:** Anthropometric and biochemical data at baseline and follow-up.

	Baseline (*n* = 25)	12 months (*n* = 25)	*p*
Age (years)	48.2 ± 11.9	—	—
BMI (kg/m^2^)	28.0 ± 5.0	27.3 ± 4.7	NS (0.090)
Waist circumference (cm)	93.1 ± 10.9	92.7 ± 11.1	NS (0.149)
Systolic blood pressure (mmHg)	130 ± 11	128 ± 11	NS (0.307)
Diastolic blood pressure (mmHg)	80 ± 9	80 ± 8	NS (0.978)
Heart rate (bpm)	73 ± 6	71 ± 7	NS (0.141)
Total cholesterol (mg/dl)	227.3 ± 35.9	201.4 ± 29.6	<0.001
LDL-c (mg/dl)	150.4 ± 30.4	130.3 ± 28.2	0.001
HDL-c (mg/dl)	59.6 ± 15.8	60.4 ± 15.9	NS (0.624)
Non-HDL-c (mg/dl)	167.8 ± 33.3	141.0 ± 29.6	<0.001
Triglycerides (mg/dl)	120.8 ± 55.5	109.1 ± 51.8	0.013
Lp(a) (mg/dl)	66.5 ± 90.5	63.9 ± 91.2	NS (0.406)
Glucose (mg/dl)	77.1 ± 25.8	83.0 ± 15.5	NS (0.165)
Creatinine (*μ*mol/l)	73.5 ± 9.2	75.7 ± 12.8	NS (0.167)
AST (U/l)	21.5 ± 6.9	21.8 ± 6.4	NS (0.777)
ALT (U/l)	18.1 ± 8.7	17.4 ± 10.2	NS (0.566)
CPK (U/l)	114.8 ± 68.3	126.0 ± 88.3	NS (0.180)
hs-CRP (mg/l)	1.74 ± 1.51	1.20 ± 1.11	0.015
Osteoprotegerin (pg/ml)	1488 ± 1241	1328 ± 1098	0.045

Data are means ± SD. BMI: body mass index; LDL-c: low-density lipoprotein cholesterol; HDL-c: high-density lipoprotein cholesterol; Lp(a): lipoprotein (a); AST: aspartate aminotransferase; ALT: alanine aminotransferase; CPK: creatin phosphokinase; NS: not significant.

## Data Availability

The study was registered with https://ClinicalTrial.org (CT Identifier: NCT04433429). The database is owned and accessible by all authors.

## Supplementary Materials

Supplementary Materials Graphical scheme of the study.
