# Supplementary material for: Lipid Profile and Vascular Remodelling in Young Dyslipidemic Subjects Treated with Nutraceuticals Derived from Red Yeast Rice
Source: Cardiovasc Ther. 2021 Apr 22;2021:5546800. doi: 10.1155/2021/5546800 (PMC8087481; doi:10.1155/2021/5546800)
Supplement: Supplementary Materials — Graphical scheme of the study. [file 5546800.f1.docx]

Puato M et al. Lipid profile and vascular remodelling in young dyslipidemic subjects treated with nutraceuticals derived from red yeast rice.

**Supplementary figure.**

Graphical scheme of the study.


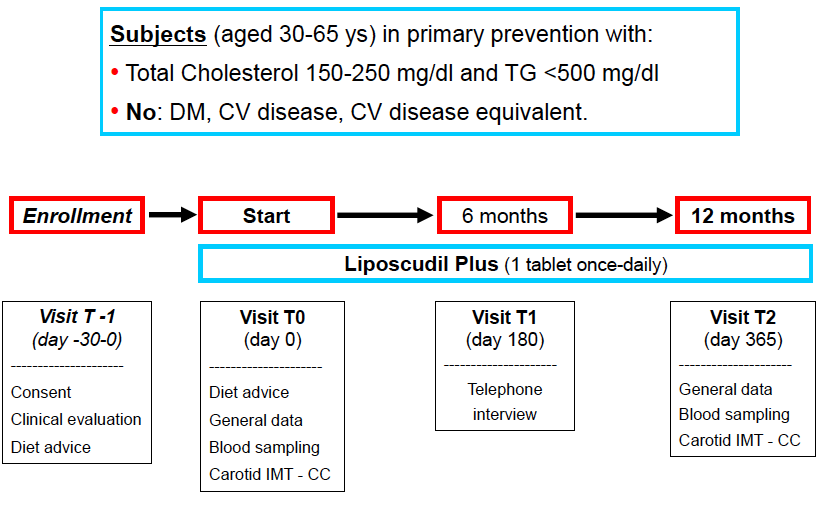


Subjects with low to moderate CV risk profile and total cholesterol levels between 150-250 mg/dl with stable dietary habits according to guidelines for CVD prevention since at least 6 months and who did meet their individual lipid goals were enrolled in this study. During the study period, patients were advised to maintain such a diet unchanged. All participants were treated with Liposcudil Plus (one pill/day, containing 333 mg of RYR, equivalent to 10 mg of Monacolin K, and 30 mg of CoQ10, Piam Farmaceutici, Italy) together with a controlled Mediterranean diet training and support.

Study assessments were performed at baseline, after 6 months, and after 12 months.

At baseline, lifestyle and medical history were recorded; anthropometric measurements were performed, and body mass index (BMI) was calculated. Systolic, diastolic blood pressure and resting heart rate were taken after three readings over a five-minute period. All subjects underwent blood sampling and carotid intima-media thickness (IMT) evaluation; carotid compliance coefficient (CC) and distensibility coefficient (DC) were derived. Women had a pregnancy test performed at recruitment. After 6 months, all participants received a phone call to verify the adherence to therapy and dietary habits. After 12 months, they underwent a new clinical visit, blood tests, IMT, CC and DC measurements.
